# Band Adhesion Causing Intestinal Obstruction Without Previous Surgery in Pregnancy: A Rare Case

**DOI:** 10.7759/cureus.81873

**Published:** 2025-04-08

**Authors:** Ritu Singh, Anand Deoraj, Monika Gupta, Deeptimayee Pathi

**Affiliations:** 1 Obstetrics and Gynaecology, Kurji Holy Family Hospital, Patna, IND; 2 Surgery, Kurji Holy Family Hospital, Patna, IND

**Keywords:** band adhesion, exploratory laparotomy, intestinal obstruction, intestinal obstruction in pregnancy, nausea and vomiting, pregnancy, previous surgery, subacute intestinal obstruction, surgery

## Abstract

Intestinal obstruction (IO) during pregnancy is a rare occurrence. Although uncommon, IO in pregnancy carries a significant risk of maternal and fetal mortality. Diagnosing IO can be challenging, as abdominal pain and vomiting in pregnant patients may be mistaken for labor pain or hyperemesis gravidarum.

Early reports strongly recommend exploratory surgery as the standard treatment once IO is diagnosed. The delivery of the baby during the same procedure may be necessary if the surgical emergency jeopardizes the pregnancy. However, this approach is not ideal if the pregnancy is not at term. In recent years, some cases have demonstrated that small bowel intestinal obstruction (SBIO) can be managed conservatively.

In our case, we successfully managed the patient conservatively before performing an exploratory laparotomy and lower segment cesarean section in the same setting to prevent maternal and fetal complications. IO without prior surgery is rare, and even more so during pregnancy. Our case highlights a rare instance of pregnancy-related IO caused by band adhesions in the absence of previous surgical history.

## Introduction

Intestinal obstruction (IO) during pregnancy is a rare occurrence, with an incidence ranging from one in 2,500 [1] to one in 16,709 deliveries [2]. Although uncommon, IO in pregnancy carries a significant maternal (3.7%) and fetal (16%) mortality risk [3]. Prior surgery is the leading cause of small bowel intestinal obstruction (SBIO) in 55% of cases [4]. Other causes include malignancy and inflammation. But in patients without previous surgery, approximately 60% of cases are attributed to congenital de novo or inflammatory adhesions, with hernia being the next most common cause. Less frequent causes of SBIO in a virgin abdomen include metastatic disease, Crohn’s disease, foreign body obstructions, gallstone ileus, and Meckel’s diverticulum [5]. 

SBIO can be challenging to diagnose, as abdominal pain and vomiting in pregnant patients may be mistaken for labor pain or hyperemesis gravidarum [3]. Early reports strongly recommended exploratory surgery as the standard treatment once IO is diagnosed, in order to prevent further morbidity and mortality for both the mother and fetus [3]. In some cases, delivery of the baby during the same procedure may be necessary if the surgical emergency jeopardises the pregnancy. However, this approach is not ideal if the pregnancy is not at term. In recent years, there have been cases demonstrating that SBIO can be managed conservatively [6,7].

In our case, we successfully managed the patient conservatively as much as possible and performed an exploratory laparotomy along with a lower segment cesarean section in the same setting to prevent both maternal and fetal morbidity and mortality. IO without prior surgery is uncommon, and since IO in pregnancy itself is rare, our case is even more exceptional. Therefore, we present the case of band adhesion-induced IO in pregnancy without previous surgery.

## Case presentation

A 27-year-old primigravida presented at 33 weeks of gestation with intermittent colicky abdominal pain, nausea, and vomiting. She did not have fever, constipation, or urinary symptoms. She had been admitted 15 days prior at 30 weeks of gestation with similar complaints, which were relieved with conservative management. She had a history of similar episodes every 5-6 months over the past 3-4 years but had been symptom-free for the last year. Her first and second trimesters were uneventful.

At the time of presentation in the emergency ward at 33 weeks of gestation, she was afebrile, with a blood pressure of 120/80 mmHg and a heart rate of 92 beats per minute. On per abdominal examination, the uterus was of 34 weeks' size. There was generalized abdominal distension with tenderness on palpation, and bowel sounds were exaggerated. Fetal heart sounds were audible, with a fetal heart rate of 136 beats per minute. A per vaginal examination revealed a closed cervix with no discharge or show. The non-stress test (NST) was reactive. Her laboratory investigation at the time of admission is shown in Table 1. Her total count was within the normal range, indicating no infection. However, her serum sodium and potassium levels were slightly low, which were corrected with intravenous fluids. 

**Table 1:** Laboratory investigation of the patient INR: International normalized ratio; ALT: Alanine aminotransferase

Lab parameter	Lab values	Reference range
Haemoglobin	11	12-16 gm/dL
Total leukocyte count	7.49x 10^3^	5.2-12.4 x 10^3 ^cells/μL
Platelet	190	140-400 x 10^3 ^cells/μL
Serum bilirubin direct/total	0.27/0.56	0.5-1.5/0-0.6 mg/dL
ALT	38	0-40 U/L
Prothrombin time/INR	14.29/1.06	11-14 sec/0.9-1.3
Random blood sugar	73	75-139 mg/dL
Urea	6.85	10-50 mg/dL
Creatinine	0.46	0.6-1.4 mg/dL
Serum sodium	132.95	135-145 meq/L
Serum potassium	3.31	3.5-5.0 meq/L

**Figure 1:**
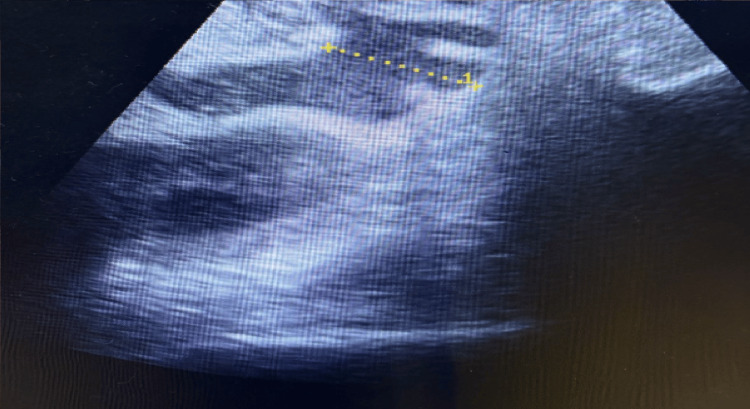
Ultrasonography of the patient The dotted yellow line shows a dilated bowel loop.

Ultrasound revealed dilated bowel loops. There was no interloop fluid or free fluid in the pelvis suggestive of subacute IO, as shown in Figure 1.

An opinion from the surgery and medicine teams was sought.

The patient was placed on a nasogastric tube (Romsons, Agra, India) and given supportive management, including intravenous fluid supplementation. After 48 hours, symptomatic management led to improvement in her condition, and she was allowed sips of water and liquid food with intermittent clamping of the nasogastric tube. Her electrolyte levels returned to the normal range. However, after 72 hours of admission, she developed high-grade fever, tachycardia, nausea, vomiting, and increased abdominal distension. She was then reinserted with a nasogastric tube for continuous drainage and kept nil per oral. Emergency laparotomy was performed along with a cesarean section in view of deteriorating maternal condition. A preterm live baby weighing 2.4 kg was delivered, and the baby cried immediately.

**Figure 2:**
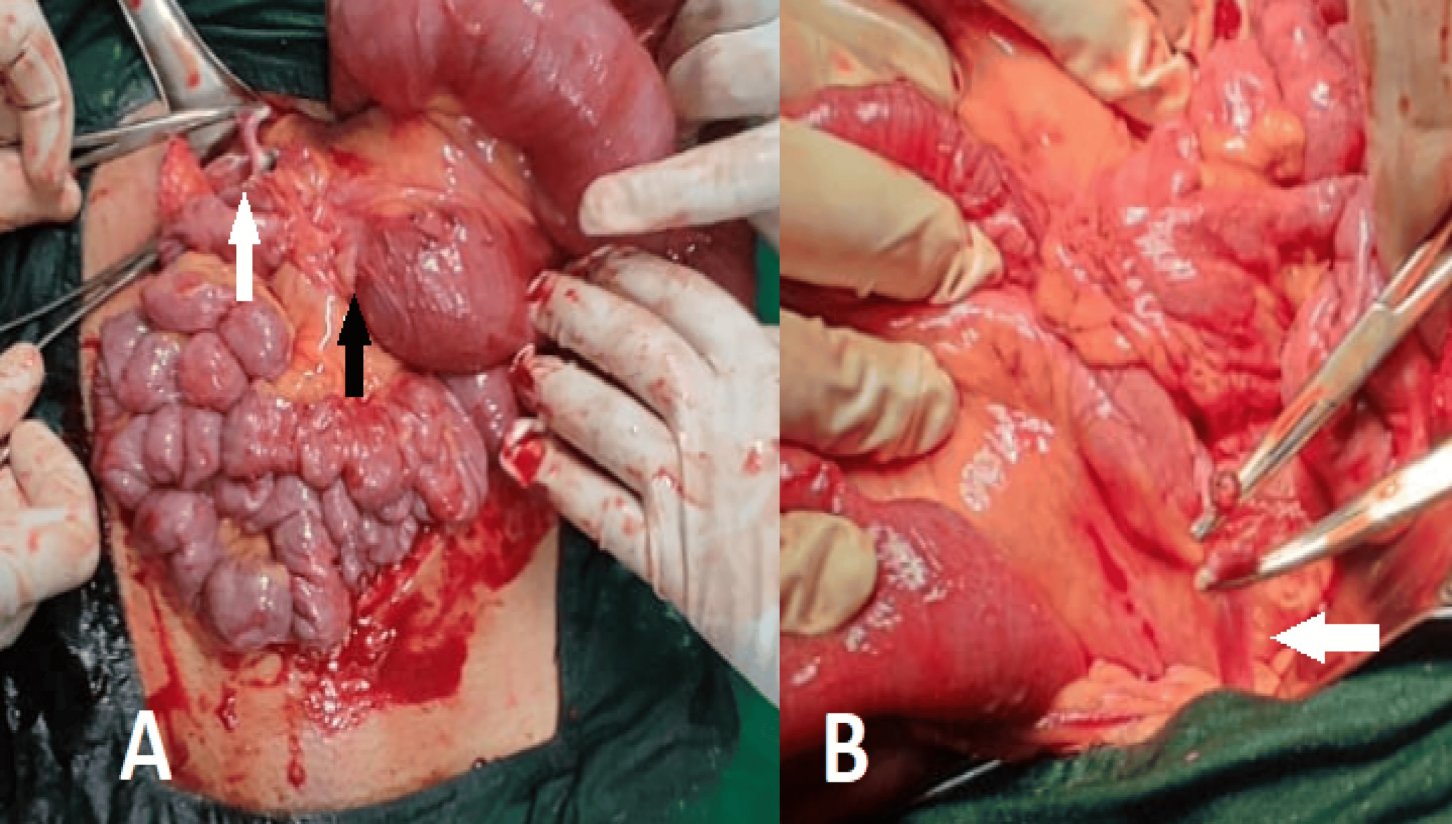
Intraoperative picture of the surgical procedure (A) The white arrow shows an adhesive band, while the black arrow shows a distended bowel loop proximal to the obstruction. (B) The white arrow shows another site of the adhesive band.

Abdominal exploration revealed distended bowel loops proximal to obstruction, as shown in Figure 2A.

Multiple adhesive bands approximately 30-40 cm from the ileocecal junction were noted. One adhesive band is shown in Figure 2B. The thickest band extended up to the anterior margin of the liver. All the bands were released, and the bowel was retrogradely decompressed. The abdomen was closed in layers. On postoperative day 1, the patient was better. The nasogastric tube was removed on postoperative day 2, and she was allowed sips of liquid fluids. She was gradually started on a normal diet by day 4 and was discharged on postoperative day 6. The patient was recalled on postoperative day 10 for suture removal, and no signs of surgical site infection (SSI) were observed.

## Discussion

Previous studies have highlighted that obstructions caused by adhesions are most common during the later stages of pregnancy [8]. This is supported by a recent review [3], which found that 52.1% of patients presented during their third trimester. Our patient also presented in the third trimester.

Abdominal pain is reported in more than 85% of pregnant women with SBIO [3]. Given the nature of pregnancy, abdominal pain is often misdiagnosed as gastroenteritis or premature labor, while vomiting could be mistakenly attributed to hyperemesis gravidarum [3]. Nausea and vomiting that persist into or begin during the third trimester should prompt further investigations to rule out IO. Our case presented with colicky pain, nausea, and vomiting.

Examination of pregnant women can be particularly challenging and nonspecific due to the presence of a gravid uterus. The enlarged uterus can obscure abdominal distension caused by SBIO. As the obstruction worsens, the uterus may contract due to irritation from the underlying condition, which can mislead obstetricians into diagnosing SBIO as early labor [3]. Our case had not gone into labor, highlighting the challenges clinicians face when diagnosing SBIO in pregnant women.

Additionally, other clinical signs such as fever, tachypnea, hypotension, and tachycardia may appear later, usually as secondary consequences of severe acidosis and infection. At this stage, the bowel may already be compromised, and it may be too late for effective surgical intervention [3]. In our case, we intervened immediately after deterioration, thereby saving both the baby and the mother.

In our patient, laboratory investigations revealed a slight electrolyte imbalance. Electrolyte imbalances commonly occur due to relative concentration changes resulting from water loss [9]. In our case, even after correcting the electrolyte imbalance, the patient showed only transient improvement before deteriorating again. This suggests that the symptoms are not due to paralytic ileus stemming from electrolyte imbalance, but rather due to a mechanical obstruction.

Ultrasound is a commonly used diagnostic tool since it does not expose the fetus to radiation. However, one study reported that only 55% of patients had ultrasound findings that corresponded with surgical findings [10]. This indicates that ultrasound is not the most reliable diagnostic method for ruling out SBIO in pregnant patients. In our patient, ultrasound revealed dilated bowel loops, with no interloop fluid or free fluid in the pelvis.

Early diagnosis and prompt intervention can significantly reduce maternal and fetal morbidity and mortality. In a recent review, 61% of patients underwent surgery within the first 24 hours of presentation, irrespective of the period of gestation, and only 21% of fetuses were delivered during surgery [3]. We chose not to intervene within the first 24 hours because each additional day in the womb provides crucial protection, supporting the development of the baby’s lungs, brain, and immune system, thereby improving the overall chances of survival and healthy outcomes after birth.

Management of the fetus during surgery depends on both the gestational age of the fetus and the maternal condition at the time of laparotomy [3]. As the mother developed fever and tachycardia in our case, it indicated the onset of sepsis, which significantly increased the risk of fetal death due to compromised maternal circulation and reduced oxygen delivery to the fetus. Since the fetus was also nearing 34 weeks of gestation, we decided to proceed with a cesarean section in conjunction with the laparotomy. If delivery is necessary during the operation, it should occur prior to addressing the obstruction [3]. In our case, the pregnancy was moderately preterm, three days short of 34 weeks, with a breech presentation, and the baby was delivered before addressing the obstruction.

Adhesions are bands of fibrous scar tissue that abnormally connect intraperitoneal organs, which are usually separate. They are classified as either congenital or acquired. Acquired adhesions commonly arise from the healing process after abdominal surgery but may also result from conditions such as malignancy, radiation, infections, or endometriosis. Congenital adhesions, on the other hand, occur without any prior abdominal disease or surgery and may originate from abnormal organ development during embryogenesis [11]. In our case, the patient did not have a history pertaining to the acquired causes of adhesions. 

However, in our case, multiple adhesive bands were identified in the small intestine. The presence of one or more bands of adhesion tissue is classified as a solitary band adhesion, while widespread, diffuse adhesions are referred to as matted adhesions [4]. Solitary band adhesion without any history of prior surgery is rare. In a study by Skoglar et al., it was found that only 12.5% of women without a history of abdominal surgery experienced obstructing solitary band adhesions [4]. From a surgical standpoint, physicians should be aware that even in patients without a history of abdominal surgery who present with signs of IO, there is a risk of obstruction and potential strangulation due to solitary band adhesions.

We did not find evidence of SSI in this patient. While open procedures have an increased risk of SSI [12], sepsis is generally not related to whether the procedure is open or minimally invasive [13].

A recently published study identified butyrylcholinesterase as a predictive biomarker for assessing the likelihood of SSI after surgery. The study found that low levels of butyrylcholinesterase on the first and third postoperative days were associated with an increased risk of developing SSIs, but not sepsis [14].

## Conclusions

Obstruction during pregnancy is rare, but when it occurs, it can lead to significant maternal and fetal mortality. It mainly occurs in the third trimester. SBIO can be particularly challenging to diagnose, as abdominal pain and vomiting in pregnant patients may be misinterpreted as labor pain or hyperemesis gravidarum. It is important to recognize that, even without a history of previous surgery, there remains a risk of IO because of solitary band adhesions.

Conservative management may be attempted until fetal viability is ensured, while remaining vigilant for any signs that would necessitate a laparotomy. The patient remained symptom-free for a period of three months. Further studies are needed for long-term follow-up of the patient.
